# Salinity Tolerance Turfgrass: History and Prospects

**DOI:** 10.1155/2013/409413

**Published:** 2013-10-03

**Authors:** Md. Kamal Uddin, Abdul Shukor Juraimi

**Affiliations:** Department of Crop Science, Faculty of Agriculture, University Putra Malaysia, 43300 Serdang, Selangor, Malaysia

## Abstract

Land and water resources are becoming scarce and are insufficient to sustain the burgeoning population. Salinity is one of the most important abiotic stresses affecting agricultural productions across the world. Cultivation of salt-tolerant turfgrass species may be promising option under such conditions where poor quality water can also be used for these crops. Coastal lands in developing countries can be used to grow such crops, and seawater can be used for irrigation of purposes. These plants can be grown using land and water unsuitable for conventional crops and can provide food, fuel, fodder, fibber, resin, essential oils, and pharmaceutical products and can be used for landscape reintegration. There are a number of potential turfgrass species that may be appropriate at various salinity levels of seawater. The goal of this review is to create greater awareness of salt-tolerant turfgrasses, their current and potential uses, and their potential use in developing countries. The future for irrigating turf may rely on the use of moderate- to high-salinity water and, in order to ensure that the turf system is sustainable, will rely on the use of salt-tolerant grasses and an improved knowledge of the effects of salinity on turfgrasses.

## 1. Introduction

Turfgrasses are among the most important industries in many countries including Malaysia because of the development in landscaping and recreation amenity [1]. Turf grass, as an important element to the landscape, serves the functions as beautification and its attractiveness are suitable for mental health, more specifically, the aesthetic effect of parks, gardens, and lawns. Turfgrass is also used to cover sports fields, such as golf, soccer and serve in the stabilization of slopes, among other purposes [2]. Turfgrasses, especially sport turf, play an important role by providing cushioning effect that could help reduce injuries to participants and improve playability.

Turfgrasses are monocot plants under the family Poaceae that act as vegetative ground cover. With its above-ground network of leaves, shoots, and stems and an extensive fibrous root system, turf grasses reduce soil erosion, remove dust and dirt from the air, release oxygen that provides a cooling effect, filter water by trapping potential groundwater pollutants, and produce safe playing surfaces for children and adults [3]. 

Salinity causes a major environmental problem limiting plant growth and productivity of both irrigated and nonirrigated lands in many areas of the world and include imposition of ion toxicities (e.g., Na and Cl), ionic imbalances, osmotic stress, and soil permeability problems [4]. In general, salt tolerance in plants is associated with low uptake and accumulation of Na, which is mediated through the control of influx and/or by active efflux from the cytoplasm to the vacuoles and also back to the growth medium [5].

Owing to added anthropogenic contributions to global warming, the rate of sea level rise is expected to increase and will have dramatic effect on crop production. In fact, global warming is one of the greatest threats now facing the planet. The oceans, which cover 71% of the Earth's surface, are currently rising at a rate of about 0.25 cm per year due to global warming [6]. However, projections for the year 2100 show great uncertainty, ranging from several centimetres to nearly a meter. The impacts of rising sea level will include increased vulnerability to storm surges and flooding of once cropped lands with salty water among many other impacts which are predicted to alter many facets of life on earth [7].

There are a number of potential turfgrass species that may be appropriate at various salinity levels of seawater. The demand for salinity-tolerant turfgrasses is increasing due to augmented use of effluent or low-quality water (sea water) for turf irrigation. This need has been exacerbated by rapid urbanisation (and associated turfgrass acreage increase) in arid/semiarid regions having intense competition for limited potable water resources [8] and in coastal areas where salt water intrusion into fresh water irrigation wells is common [9]. A new generation of turf varieties allows landscape development in saline environments [10, 11]. Such type of several grasses has now been developed and selected to produce plant varieties that can be utilized as turf. These turfs are ideal in environments in which salinity is a problem or where limited or no fresh water is available for irrigation.

## 2. Natural and Taxonomic Distribution of Turfgrasses

The turfgrass species are in the family Poaceae which was formerly known as Gramineae under the class Monocotyledoneae. More than 800 genera comprising over ten thousand species belong to the Poaceae [12]. Each species may contain a number of cultivars or varieties. Most cultivars are produced by hybridization followed by natural selection and also artificial selection. In consideration of life cycle, annual and perennial turfgrasses are available throughout the ecosystems [13]. 

Differences in ecological adaptation of turf determine their obvious geographical distribution over the climatic regions of the world. Bermudagrass, Cowgrass, Serangoongrass, Zoysiagrass St. Augustinegrass, Bahiagrass, Seashore Papalum, and Centipedegrass are highly appreciated as tropical warm season turfgrasses. On the other hand, Kentucky bluegrass, Rough Bluegrass, Canada Bluegrass, Annual Bluegrass, and Annual Ryegrass have established their position in the list of cool season turfs [14]. 

## 3. Morphology of Turfgrass Species 

Most grasses are established by seeding, but, in some cases, they can also be established vegetatively using sod, sprigs, stolons, or plugs. Turfgrasses have an extensive fibrous root system. It is common for grasses to have several tons of roots per acre. The bulk of the root system is in the top 10 to 15 cm of soil. They may have some roots that grow down several feet into the soil. The three major types of stems associated with turfgrass are the crown, the flowering culm, and lateral or creeping stems. The crown, the principal meristematic region, is an unelongated stem. At reproductive stage, it produces an elongated stem, which is called the flowering culm. Some turfgrass species have lateral or creeping stems called stolons and rhizomes. These stems elongate horizontally from the crown of the parent plant. Stolons grow along the surface of the ground, while rhizomes grow beneath the surface. Shoots and roots form at nodes on the horizontal stems [14].

A leaf of turfgrass is divided into two major parts: the sheath and the blade. The blade is the upper, relatively flat part of the leaf. The sheath is the cylindrical portion of the leaf that surrounds the culm or young leaves. The sheath margin (the area where the two edges come together around the culm) can be used for identification. These two edges or margins can be open (not touching), closed (seamless), or overlapping. The sheaths are rolled around each other and support the leaf blades, holding them above the ground so that they can intercept sun light. A collar is the growing area or band that divides the sheath and leaf blade. Auricles are outgrowths that arise on each side of the collar. They can be short and blunt, long and clasping, or absent. Another feature, the ligule, is an outgrowth that arises at the inside junction of the sheath and leaf blade. The inflorescence, produced at the top of the culm, is the flowering part of a grass plant and is where seeds are formed [15]. 

## 4. General Aspects of Salinity Problems in Plants

Salinity causes major environmental factors limiting plant growth and productivity in many areas of the world. Salinity is one of the most important abiotic stresses widely distributed in both irrigated and nonirrigated areas of the world. Plants that grow on saline soils are confronted with soil solutions exhibiting a wide range of concentrations of dissolved salts. Concentrations fluctuate because of changes in water source, drainage, evapotranspiration, solute availability, and hydrostatic pressures. The leaves of glycophytic plants cannot retain high levels of salt without injury. In addition to the osmotic effect of concentrated solutes, there are ionic effects that arise from the specific composition of the solute flowing through plant tissues. Internal excesses of particular ions may cause membrane damage, which interferes with solute balances or causes shifts in nutrient concentrations. Some specific symptoms of plant damage which may be recognized especially in the leaves are colour change, tip-burn, marginal necrosis, and succulence [16, 17].

## 5. Salinity in Water and Soil

The technical term for saltiness in a solution is salinity. Different types of units have been used for salinity level expression. These are molarity (M), milli molarity (mM) (based on molecular weight of the salt), micro Siemen (*μ*S cm^−1^), milli Siemen (mS m^−1^), deci Siemen (dS m^−1^) (based on electrical conductivity), and % salt (based on percent concentration of the salt). Among these, mM, dS m^−1^, and % salt concentrations are most generally used. Approximately 58.45 mg NaCl per litre is 1 mM solution of NaCl, and 640 mg NaCl per litre is equivalent to the EC value of 1 mmhos cm^−1^ or 1 dS m^−1^ [18]. Therefore, 1 dS m^−1^ salinity is equivalent to 11 mM salt solution. When salinity is expressed in terms of % concentration of salt solution it is estimated that 1% concentration is equivalent to 16 dS m^−1^ [19].

The salinity of a solution is measured using an electrical conductivity meter (EC meter), whereas *in situ* mud and soil salinity are measured using a conductivity probe [18]. Electrical conductivity of a solution (EC), expressed in dS m^−1^ at 25°C, is recommended as a salinity index by US Salinity Laboratory [20]. According to the description of the US Salinity Laboratory (1954) the saturation extract of a saline soil has an electrical conductivity (EC) greater than 4 dS m^−1^ and an exchangeable sodium percentage (ESP) < 15. Although the pH of saline soils can vary over a wide range, it is usually around neutrality, with a tendency toward slight alkalinity (less than 8.5). Saline soils with an ESP of greater than 15 are termed saline-alkaline soils (or saline-sodic soils), have high pH values, and tend to become rather impermeable to water and air when the soluble salts are removed by leaching [21]. Although NaCl is predominant [22, 23], ionic constituents include varying proportions of chlorides, sulphates, bicarbonates, carbonates, and occasionally nitrates and borates of Na, K, Ca, and Mg [16]. Seawater contains generally Na, Mg, SO_4_, Ca, and HCO_3_ at 77.4, 17.6, 9.2, 3.4, and 0.4 meq L^−1^, respectively [24].

## 6. Tuning Mechanisms in Plants under Saline Environment

Regulation of ion transport is one of the most important factors responsible for salt tolerance of plants. Membrane proteins play an important role in selective distribution of ions within the plant or cell [25]. The membrane proteins are involved in cation selectivity and redistribution of Na^+^ and K^+^ [26]. These proteins are: (a) primary H^+^-ATPases which generate the H^+^ electrochemical gradient that drives ion transport, (b) Na^+^/H^+^ antiports in the plasma membrane for pumping excess Na^+^ out of the cell, (c) Na^+^/H^+^ antiports in the tonoplast for extruding Na^+^ into the vacuole, and (d) cation channels with high selectivity for K^+^ over Na^+^. It is well established that Na^+^ moves passively through a general cation channel from the saline growth medium into the cytoplasm of plant cells [21, 27], and the active transport of Na^+^ through Na^+^/H^+^ antiports in plant cell is also evident [28]. Energy-dependent transport of Na^+^ and Cl^−^ into the apoplast and vacuole can occur along with H^+^ electrochemical potential gradients generated across the plasma membrane and tonoplast [29]. The tonolast H^+^ pumps (H^+^-ATPase and H^+^-pyrophophatase) also play a vital role in the transport of H^+^ into the vacuole and generation of proton (H^+^) which operates the Na^+^/H^+^ antiporters [27, 30]. 

Within the general hypothesis of a NaCl-induced disturbed nutrition, the dominant specific hypothesis has clearly been that of “ion excess,” that is, the idea that Na^+^ and/or Cl^−^ rise to toxic levels in the shoot, eventually to high levels in the cytoplasm leading directly to metabolic inhibition [31]. There are three major constraints for plant growth on saline substrates. 

**Figure 1:**
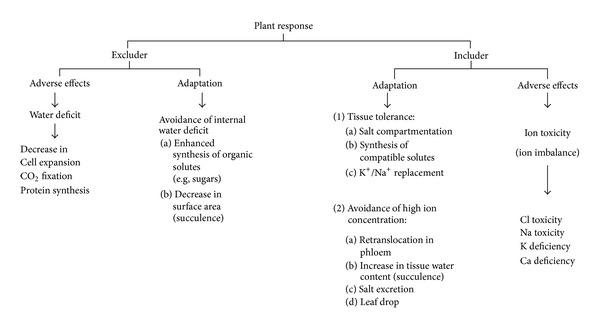
Adverse effects of salinity and possible mechanisms of adaptation (adapted from [21]).

Water deficit (drought stress) arising from the low (more negative) water potential of the rooting media;

- Ion toxicity associated with the excessive uptake mainly of Na^+^ and Cl^−^.
- Nutrient imbalance by depression of mineral nutrient uptake, Ca^2+^ in particular.
- The effects of salinity and possible mechanisms of adaptation by plants are summarized in Figure 1.

## 7. Salt-Tolerant Turfgrass Species 

Relative salinity tolerance among turfgrass species and cultivars has been associated with restriction of saline ion accumulation in shoots [32]. Many different criteria have been used to measure salinity tolerance of turfgrass, such as shoot and root weight, shoot weight reduction relative to a nonsaline control, visual scores of salinity injures such as leaf firing, plant survival, and seed germination [33]. Also the EC at 25 or 50% shoot and root growth reduction has been used for relative tolerance rankings [34].

*Paspalum vaginatum* is one of the most salt-tolerant turfgrasses where sea water or any type of reclaimed/recycled water can be used for irrigation [35]. An estimated relative salinity tolerance of *Paspalum vaginatum* for 50% decrease in growth is 25 dS m^−1^ [36]. Most *Paspalum vaginatum* exhibited halophytic responses to salinity and some could tolerate sea water salinity [37, 38]. *Paspalum vaginatum* has the potential to be one of the most environmentally compatible turfgrasses in the near future [39, 40]. Bermudagrass also exhibits good tolerance to salty water [41]. Fifty percent shoot growth reduction for Bermudagrass cultivars and accessions has been reported at salinity levels of 24 and 33 dS m^−1^ [42]. Zoysiagrass having good salinity tolerance has recently been developed. Zoysiagrass has long been considered a salinity tolerant turfgrass and has been reported as equivalent in salinity tolerance to a highly salt-tolerant seashore paspalum [43, 44]. 

## 8. Effect of Salinity on Turfgrass Morphology 

Osmotic adjustment under increased salinity occurred in Seashore paspalum, St. Augustine grass, Bermuda grass, Manila grasses and Japanese lawn grass concurrent with increased shoot Na^+^ and Cl^−^ concentrations, decreased shoot K^+^ concentration, and decreased shoot succulence [45–48]. Osmotic adjustment and maintenance of positive turgor under salt stress occurred in Seashore paspalum Turfgrasses may exclude saline ions in several ways: via compatible solute accumulation in association with ion compartmentalization, and excretion [33]. In Bermudagrass and other turfgrass species it was found that proline and glycine betaine levels increased as salinity increased [49–51]. Most of the salt-tolerant plants can still function by maximizing water uptake and turgor pressure meaning that water relations are important for negating salinity stress. Salt-tolerant turfgrasses have the ability to minimize the detrimental effects by producing a series of anatomical, morphological, and physiological adaptations. However, salinity causes lower osmotic potential, loss of turgor potential, ion toxicity, and nutritional disturbances [52]. Relative salinity tolerance is generally quantified as the salt level resulting in a 50% shoot growth reduction [34]. 

Zoysiagrass cultivars having good salinity tolerance have recently been developed; these have a high degree of salt gland activity [43]. The two-phase growth response curve has three essential parameters used for classifying plant salinity tolerance [53–55]: (i) threshold EC_*e*_, the maximum soil salinity that does not decrease yield below that obtained under nonsaline conditions; (ii) the slope of the section where increasing salinity reduces growth, which is represented as the yield decline per unit increase in salinity beyond the threshold EC_*e*_; and (iii) the EC_*e*_ related to 50% growth reduction. 

## 9. Effect of Salinity on Physiological Process of Turfgrass

Plants osmotic adjustment subjected to salt stress can occur by the accumulation of high concentration of either inorganic ions or low molecular weight organic solutes. Although both of these play a crucial role in higher plants grown under saline conditions, their relative contribution varies among species, among cultivars, and even between different compartments within the same plant [56]. The detrimental salinity effects on plants include growth suppression, lower osmotic potential, loss of turgor potential, ion toxicity, and nutritional disturbances [52]. 

References [45, 46] also demonstrated that, as salinity increased, plant K levels decrease and to a lesser degree there is a decrease in Ca, Mg, and P.

Some selections of Seashore paspalum can tolerate undiluted seawater under the correct management regimes. Seawater has an EC of 54 dS m^−1^ (34 560 mg/L), and these new salt-tolerant varieties provide an opportunity to use very brackish sources of water though a high level of management is required [35].

Salinity effects on turfgrass growth have been summarised by [8] as

 reduced water uptake due to osmotic stress;
 reduced nutrient uptake, for example, K may be depressed by absorption of Na;
 root biomass may increase to improve water-absorbing ability; and
 Na and Cl reduce growth by interfering with photosynthesis.

Some cool and warm season turfgrasses were classified in Table 1 on the basis of salt tolerant level.

## 10. Conclusion

The development of turfgrass industry in the coastal areas is challenging due to scarcity of fresh water for irrigation. The relative salinity tolerance of turfgrass root growth, shoot growth, and leaf firing was closely associated with salinity tolerance of the grasses. The different species of grasses were grouped for salinity tolerance on the basis of 50% shoot and root growth of reduction, leaf firing, and turf quality with increasing salinity. The use of halophytes for rehabilitation and reclamation of salt-affected lands has proven to be feasible if certain precautions are taken. Plantations of halophyte species are justified when they can make areas productive. The soil/water management practices to provide adequate rainage and other soil-related aspects are critical factors in using saline water for irrigating halophytes. There is a need for developing the proper agromanagement and conditions to maximize the productivity of these known economical halophytic species.

## Figures and Tables

**Table 1:** Estimated salt tolerance of common cool and warm season turfgrass [8, 56, 57].

Cool season turfgrass	Rating	Warm season turfgrass	Rating
*Puccinellia spp.* (Alkaligrass)	T	*Paspalum notatum* Flugge (Bahiagrass)	MS
*Poa annua* L. (Blue grass)	S	*Cynodon dactylon* (Bermuda “Tifdwarf”)	MS
*Lolium multiflorum* (Annual ryegrass)	MS	*Cynodon dactylon* (Bermuda “Satiri”)	MT
*Festuca rubra* L. spp. (Chewing fescue)	MS	*Bouteloua gracilis *(H.B.K) (Blue Grama)	MT
*Agrostis tenuis* (Colonial bent grass)	S	*Bouteloua dactyloides* Nutt. (Buffalo grass)	MT
*Agrostis palustris* (Creeping bent grass)	MS	*Eremochloa ophiuroides *(Centipedegrass) Munro	S
*Festuca rubra* L. spp. rubra (Creeping red fescue)	MT	*Paspalum vaginatum* (Seashore paspalum)	T
*Agropyron cristatum* (Fairway wheat grass)	MS	*Stenotaphrum secundatum* (St. Augustine)	T
*Festuca longifolia* Thuill. (Hard fescue)	MT	*Zoysia japonica* (Japanese lawn grass)	T
*Poa pratensis* L. Kentucy (Blue grass)	MS	*Zoysia matrella* (Manila grass)	MT
*Lloium perenne* L. (Perennial rye grass)	S	*Zoysia tenuifolia* (Korean grass)	MS
*Festuca arndinacea* Schreb. (Tall fescue)	MT	*Digitaria didactyla* Wild (Serangoongrass)	MT

The rating is based on soil salt test levels, sensitive <3 dS m^−1^, moderate sensitive 3–6 dS m^−1^, moderate tolerant 6–10 dS m^−1^, and tolerant >10 dS m^−1^.
